# Comparing the Efficacy of Myo-Inositol Plus α-Lactalbumin vs. Myo-Inositol Alone on Reproductive and Metabolic Disturbances of Polycystic Ovary Syndrome

**DOI:** 10.3390/metabo13060717

**Published:** 2023-05-31

**Authors:** Zdravko Kamenov, Antoaneta Gateva, Simona Dinicola, Vittorio Unfer

**Affiliations:** 1 Department of Internal Medicine, University Hospital “Alexandrovska”, Clinic of Endocrinology and Metabolism, Medical University, 1431 Sofia, Bulgaria; 2 The Experts Group on Inositol in Basic and Clinical Research (EGOI), 00161 Rome, Italy; 3 Systems Biology Group Lab, 00161 Rome, Italy; 4 UniCamillus—Saint Camillus International University of Health Sciences, 00131 Rome, Italy

**Keywords:** polycystic ovary syndrome, myo-inositol, α-lactalbumin, hyperandrogenism

## Abstract

Despite the beneficial effect of myo-inositol on metabolic, hormonal, and reproductive parameters of polycystic ovary syndrome (PCOS) patients, 28% to 38% could be resistant to this treatment. The combination with the milk protein α-lactalbumin can be a useful therapeutic approach to overcome inositol resistance and achieve ovulation in these women. This open-label prospective study aimed to compare the effects of supplementing myo-inositol plus α-lactalbumin vs myo-inositol alone on reproductive and metabolic abnormalities in PCOS. A total of 50 anovulatory women with a PCOS diagnosis were randomly assigned to receive myo-inositol alone or a combination of myo-inositol and α-lactalbumin for three months. Anthropometric measures, hormonal levels, and menstrual cycle duration were collected at baseline and after treatment. The therapy with myo-inositol plus α-lactalbumin improved both ovulation rate and menstrual cycle duration more than myo-inositol alone. The body weight was significantly reduced in women receiving myo-inositol plus α-lactalbumin, while patients in the myo-inositol group experienced no change. In addition, the improvement of hyperandrogenism was more prominent in patients treated with myo-inositol plus α-lactalbumin. The benefits of associating myo-inositol and α-lactalbumin clearly make this combination a true edge in the management of PCOS.

## 1. Introduction

Inositols have captured the interest of researchers and physicians alike in the past 20 years, having shown remarkable potential in the fields of gynecology and endocrinology. In total, inositol is made up of a group of nine polyol isomers (cis-, epi-, allo-, myo-, neo-, scyllo-, L-chiro-, D-chiro, and muco-inositol), of which the two most common, myo-inositol (myo-Ins) and D-chiro-inositol (D-chiro-Ins), account for the vast majority observed in nature. Myo-Ins can be introduced into the body through diet, with the average person consuming 1 g of inositols per day with foods such as cereals, legumes, oil seeds, and nuts providing reliable sources. However, the body can synthesize most of the daily requirement of myo-Ins, amounting to 4 g per day. Endogenously, the synthesis of myo-Ins begins with glucose-6-phosphate, which is isomerized by the D-3-myo-inositol-phosphate synthase enzyme (inositol synthase, Ino1, or MIPS1) to inositol-3-phosphate (InsP3). This latter is subsequently dephosphorylated through the use of inositol monophosphatase-1 to release free myo-Ins. Alternatively, free myo-Ins can be released through the dephosphorylation of various inositol phosphates, namely InsP3 and InsP2. It has been long established that myo-Ins works as a mediator of insulin signaling and, as such, plays important functions in regulating glucose homeostasis, mainly by stimulating intracellular glucose uptake [1,2]. 

Furthermore, in women, myo-Ins acts as the second messenger of several endocrine signals, namely the follicle-stimulating hormone (FSH) and the thyroid-stimulating hormone (TSH). Due to its role in FSH regulation, myo-Ins is essential for ensuring both the proper maturation and the quality of the oocyte participating in several biochemical pathways [3,4,5]. As such, myo-Ins modulates FSH-mediated anti-Mullerian hormone (AMH) production, therefore playing a pivotal role in determining transport in the oviduct as well as ensuring the good quality of embryos. This gives a rationale for its ever-increasing use in assisted reproduction technologies (ART), in combination with gonadotropins [6], as well as to support physiological pregnancy [7,8].

A large number of studies have demonstrated that treatment with myo-Ins significantly improves ovarian function and fertility, mainly in those patients with a diagnosis of polycystic ovary syndrome (PCOS) [9,10,11]. PCOS is the most common endocrine disorder affecting women of reproductive age, with approximately 1 in 10 women thought to face PCOS before menopause. It is currently defined using the Rotterdam criteria, which require two of the following criteria: (1) chronic ovulation disorder (oligo-ovulation, anovulation, and amenorrhea); (2) presence of polycystic ovaries as identified through the use of an ultrasound examination; and (3) hyperandrogenism. The use of myo-Ins in patients with PCOS decreases the severity of several common symptoms, namely hyperandrogenism, acne, and hirsutism [12,13,14], and positively affects metabolic parameters (insulin resistance, lipid profile, etc.). The modulation of various hormonal parameters, deeply involved in ovulation and reproductive functionality [15,16], has made myo-Ins supplementation a novel and effective approach to improve spontaneous ovulation [9,10,11,12,13,14,15,16,17] or to induce ovulation in oligo- and anovulatory women [18,19,20,21]. However, unfortunately, not all patients respond in the same manner and with the same effectiveness to inositol supplementation with regard to ovulation. Several authors indicated such failure as “inositol resistance”, which may vary from 28% to 38% among different studies [11,22,23]. The cause of inositol resistance is not yet well understood. In fact, in most of the trials evaluating the rate of myo-Ins-induced ovulation, the differences in terms of hormonal and metabolic profiles between responders and non-responders have not been extensively assessed. 

Keeping in mind the complex and multifactorial pathogenesis of PCOS, which includes central dysregulation of the hypothalamic “pacemaker” mechanisms, insulin resistance, and low follicular and oocyte quality, it is understandable that different treatments have been proposed and explored.

Several studies proved the effectiveness of myo-Ins in counteracting the signs of PCOS pathogenesis [24]. However, during the treatment, differences in myo-Ins plasmatic bioavailability seem to be involved in the phenomenon of inositol resistance. 

α-lactalbumin (α-LA) is a protein naturally found in mammalian milk (20–25% of whey) that has a role in improving myo-Ins plasmatic availability. α-LA acts not only as a nutrient but also as a factor enhancing the intestinal absorption of other nutrients, such as vitamins and microelements. Due to its water solubility and heat stability, α-LA finds application as a supplemental protein in various food products, beverages, or in various product formulations such as gels, emulsions, and foams. It has further potential applications in the formulation of milk for infants as an alternative to protein sources that usually derive from bovine sources. This is due to α-LA’s high concentration of essential amino acids, which therefore allows for reduced protein content, thus minimizing the risk of excessive weight gain in the infant. Furthermore, α-LA improves the gastrointestinal side effects typically associated with regular formula, with the α-LA enriched formula being similar to those of human milk and much lower than the standard formula [25]. When orally administered, α-LA does not precipitate in the gastric environment but passes unchanged through the stomach and becomes a substrate of intestinal pancreatic enzymes, providing bioactive peptides that are responsible for multiple biological actions [26]. This biological activity has been further studied, with α-LA showing applications in modulating neurological function in applications towards sleep or depression, in addition to a diverse range of pathologies such as sarcopenia, mood disorders, seizures, and cancer. 

To evaluate the effect of α-LA on the bioavailability of myo-Ins a clinical study was conducted in healthy subjects and revealed that, although the peak concentration at 180 min was similar when myo-Ins was supplemented in monotherapy and in combination with α-LA, the combined administration in a single dose led to significantly higher plasma concentrations of myo-Ins [26]. The same study group demonstrated that the presence of digested α-LA improved myo-Ins intestinal absorption in Caco-2 cells, possibly due to changes in tight junctions’ permeability [26]. Another possible mechanism explaining the improved absorption of myo-Ins might involve the stimulation of glucagon-like peptide-2 (GLP-2) secretion from gut endocrine cells induced by α-LA hydrolysate. The authors observed such a mechanism in rats [27], and they also proved it may enhance small intestine absorption [28]. In specific conditions, α-LA can also work as a suitable carrier to increase the passage of molecules across the intestinal barrier.

With this in mind, the aim of the present study was to compare the efficacy of supplementing myo-Ins in combination with α-LA (myo-Ins + α-LA) vs myo-Ins alone on reproductive outcomes (mainly ovulation) and metabolic disturbances in patients with PCOS. One of the goals of this trial is paving the way for larger studies to fully interrogate the phenomenon of inositol resistance. 

## 2. Materials and Methods

### 2.1. Patients and Treatments

In the present open-label randomized prospective study, we included 60 anovulatory patients of reproductive age (18–45 years) with a diagnosis of PCOS according to the Rotterdam criteria. They were assessed for eligibility from 2019 to 2022 at the Department of Internal Medicine of the Medical University of Sofia (Sofia, Bulgaria) and at the Clinic of Endocrinology of the University Hospital “Alexandrovska” of Sofia (Sofia, Bulgaria). 

The inclusion criteria was PCOS diagnosis according to the Rotterdam Criteria.

The exclusion criteria were:

− Other conditions causing ovulatory disorders and/or androgens hyperproduction include hyperprolactinemia, hypothyroidism, adrenal hyperplasia, and Cushing syndrome.
− Hormonal and/or pharmacological treatments in the previous 3 months that could interfere with ovulation.
− Drastic changes in bodyweight (>5%) during the last 3 months.
− Treatment with food supplements containing inositols (myo-Ins, D-chiro-Ins) in the previous 3 months.
− Pharmacological treatment with pioglitazone or metformin in the previous 3 months.
− Diseases related to digestive absorption

The trial was approved by the Ethics Committee of University Hospital “Alexandrovska” of Sofia (Sofia, Bulgaria) (protocol 359/19 December 2018).

The design of the trial met the Declaration of Helsinki, and all the included patients signed an informed consent form for their participation in the study. 

All the patients fulfilling the inclusion/exclusion criteria were randomly assigned to one of the two therapeutic groups. In order to achieve equal sample sizes, block randomization was used. Group 1 received 2 g of myo-Ins alone (Inofolic^®^, Lo.Li. Pharma Co., Rome, Italy), while group 2 was supplemented with a combination of 2 g of myo-Ins and 50 mg of α-LA (Inofolic^®^ HP, Lo.Li. Pharma Co., Rome, Italy). 

All the patients took two sachets per day (morning and evening away from meals) for three months, and then they came for the post-treatment evaluation.

The two groups did not differ in lifestyle intervention (diet and exercise), and all women kept a sedentary habit. 

The primary outcome was the induction or restoration of ovulation, while, as secondary outcomes, we evaluated the improvement of insulin resistance by oral glucose tolerance test (OGTT) and HOMA index, hyperandrogenism (quantifying total testosterone, acne, and hirsutism), hormonal parameters by measuring luteinizing hormone (LH), FSH, LH/FSH), and body mass index (BMI).

The ovulation was detected using ultrasound folliculometry and, when not possible (because of COVID-19 restrictions), by self-use ovulation test strips measuring the levels of LH in the urine.

### 2.2. Patients’ Evaluations and Plasma Analyses

At the enrollment and after three months of supplementation (at the early follicular phase—on days 2–5 of the spontaneous or drug-induced menstrual cycle), we performed:

1. Anthropometric measurements (height, weight, waist-to-hip ratio) and BMI
2. Hirsutism evaluation using the Ferriman-Gallwey (FG) score (FG score ≥7 points was regarded as hirsutism)
3. Questions regarding the menstrual cycle of the previous 3 months
4. Ovarian ultrasound to verify the absence or presence of polycystic ovaries
5. Blood analyses:
  - OGTT at 0, 60, and 120 min
  - Total testosterone
  - LH
  - FSH
  - 17β-estradiol (E2)
  - Dehydroepiandrosterone sulphate (DHEAS)
  - Androstenedione
  - 17(OH) progesterone

For the OGTT, we used 75 g of glucose with the measurement of immunoreactive insulin (IRI) [electrochemiluminescence method (ECLIA, Elecsys 2010, measuring range 0.2–1000 mcU/mL)]. Testosterone levels were measured using an electrochemiluminescence (ECLIA) method by Elecsys 2010 (analytical sensitivity: 0.069 nmol/L (0.02 ng/mL). To measure DHEAS levels, an electrochemiluminescence (ECLIA) method was used by analytics Elecsys 2010 (sensitivity 0.003 mcmol/L). Androstenedione levels were measured by a solid-phase enzyme-linked immunosorbent assay (ELISA), based on the principle of competitive binding [analytical sensitivity 0.019 ng/mL (0.663 nmol/L)]. Levels of 17-OH-progesterone were measured by a solid-phase enzyme-linked immunosorbent assay (ELISA), based on the principle of competitive binding [analytical sensitivity 0.034 ng/mL (0.103 nmol/L)]. Estradiol levels were measured by an immunochemiluminescent test [analytical sensitivity 7.9 pg/mL (29 pmol/L)]. LH levels were measured by an immunochemiluminescent test (analytical sensitivity 0.07 IU/L). FSH levels were measured by an immunochemiluminescent test (analytical sensitivity of 0.03 IU/L).

### 2.3. Statistics

The data were processed using the statistical package SPSS 23.0 (IMBTM). The level of significance for rejecting the null hypothesis was *p* < 0.05. The following statistical methods were applied: descriptive analysis, variation analysis, Kolmogorov–Smirnov’s one-sample non-parametric test, Student’s *t*-test for 2 independent samples, Mann–Whitney’s non-parametric U test for 2 independent samples, correlation analysis, binary logistic regression, and the Chi-square test. 

Data are presented as mean ± SD, except for data analyzed via non-parametric tests which are reported as median [25th percentile–75th percentile].

## 3. Results

In the present study, we included 60 women responding to the Rotterdam criteria for PCOS diagnosis. We randomly assigned such patients to one of the two study groups: 30 women were included in group 1 taking myo-Ins alone, while 30 women in group 2 took the combination myo-Ins + α-LA. During the study, 10 patients dropped out (primarily because of the COVID-19 pandemic), and we performed the final assessment at the follow-up on 50 patients—21 from group 1 and 29 from group 2. 

The baseline characteristics of the groups are shown in Table 1. The two groups were similar in age, menstrual cycle duration, FG score, androgen levels, and HOMA-index, while the prevalence of hirsutism, hyperandrogenaemia, and insulin resistance varied by about 10%. 

By stratifying the patients according to PCOS phenotypes, 58% of them belonged to phenotype A of PCOS (full-blown syndrome including hyperandrogenism, clinical or biochemical ovulatory dysfunction, and polycystic ovaries); 6% to phenotype B (including hyperandrogenism and ovulatory dysfunction); 12% to phenotype C (including hyperandrogenism and polycystic ovaries); and 24% to the D phenotype (non-hyperandrogenic PCOS including ovulatory dysfunction and polycystic ovaries). Phenotype rates were consistent across both treatment groups. 

After three months of supplementation, we found a significantly higher rate of ovulation in women treated with myo-Ins + α-LA with respect to myo-Ins-treated patients (79.3% vs 47.6% of the patients; *p* = 0.019).

An improvement in menstrual cycle duration was observed in 84.0% of women treated with myo-Ins + α-LA while only 55.6% of patients receiving myo-Ins improved their menstrual cycle duration (*p* = 0.044). Moreover, the patients in the myo-Ins + α-LAgroup exhibited a shorter post-treatment menstrual cycle duration (33.2 ± 17.2 vs 45.3 ± 25.1 days; *p* = 0.049). The mean menstrual cycle duration was reduced to 17.8 ± 19.3 days in myo-Ins + α-LA patients vs 4.7± 7.5 days in the myo-Ins patient group (*p* = 0.05). At three months, the mean menstrual cycle duration in those patients reporting improvements was 30.2 ± 3.9 days in the myo-Ins + α-LA group and 31.3 ± 3.6 days in the myo-Ins group (not significant). New pregnancies occurred in one patient in the myo-Ins group and another in the myo-Ins + α-LA. Table 2 shows the outcomes after the three-month therapy. Statistical significance was determined by the Chi-square test.

Baseline hormonal and anthropometric parameters reported no significant difference, as did insulin resistance indices in patients who improved their menstrual cycle after treatment in comparison to the non-responders. We obtained the same results when we compared responders and non-responders within treatment groups or when we considered responder patients that had improvements in either menstrual cycle, hyperandrogenemia, hyperandrogenism, or weight. Patients who responded to the treatment with an improved menstrual cycle had significantly shorter baseline menstrual cycle duration (49.7 ± 17.4 vs. 65.9 ± 31.2 days; *p* = 0.036). 

The 29 patients in the myo-Ins + α-LA group experienced a significant reduction of their body weight (−2.2 ± 3.2; from 70.60 ± 17.70 to 68.45 ± 15.76; *p* = 0.001) in comparison to the 21 patients on myo-Ins alone (0.02 ± 0.96; from 65.03 ± 10.99 to 65.05 ± 10.95; *p* = 0.929); between treatment groups (*p* = 0.05). Patients that restored a normal menstrual cycle during treatment experienced an even bigger difference in weight change (−2.35 ± 3.2 kg vs. 0.10 ± 1.10 kg; *p* = 0.025). 

In addition, clinical signs of hyperandrogenism greatly improved after the combined treatment. Hirsutism (75% vs. 33.3%; *p* = 0.034) and also the mean FG score decreased (−1.24 ± 1.4 vs. −0.43 ± 1.4 points; *p* = 0.046) in patients treated with myo-Ins + α-LA rather than with myo-Ins alone. The prevalence of acne was very low at the baseline visit, and no change occurred during the treatment period. No patients exhibited androgenetic alopecia at baseline or at the follow-up visit. 

Moreover, in the myo-Ins + α-LA treated group, the more hyperandrogenic the patients were at baseline, the more their hyperandrogenemia benefited from the treatment with respect to the myo-Ins group (55.0% vs. 46.7%; *p* = 0.44). 

Furthermore, insulin resistance improved in the myo-Ins + α-LA group with respect to myo-Ins (61.5% vs. 44.4%; *p* = 0.361), although without reaching statistical power, as did the modifications in the HOMA-index (−0.15 ± 1.7 vs. −0.19 ± 1.2; *p* = 0.94). 

No patients in both groups reported side effects.

## 4. Discussion

Inositols—mainly myo-Ins—are involved in many biochemical pathways within oocytes, having a role in oocyte maturation, fertilization, implantation, and post-implantation development. Despite the efficacy of myo-Ins on metabolic, hormonal, and reproductive signs of PCOS, not all the supplemented patients succeed in ovulation. Several authors regard such failure as “inositol resistance” [19]. This phenomenon ranges from 28% to 38% among different studies. The reason for this resistance is still unclear, but it could correlate to metabolic and hormonal dysregulations, including obesity, insulin resistance, and hyperandrogenemia, or to differences in the bioavailability of myo-Ins. As a matter of fact, the available data suggest that inositol resistance is more likely in moderate/severe obese, insulin-resistant, and hyperandrogenic women with PCOS [19]. 

Montanino Oliva et al. [29] performed a clinical study on myo-Ins-resistant patients. In such women, plasma levels of myo-Ins did not increase, thus raising the question about the role of myo-Ins bioavailability in non-responder individuals. Various factors may affect myo-Ins bioavailability, including intestinal absorption, transport from plasma into tissues, endogenous synthesis and catabolism, kidney excretion, iatrogenic depletion, etc. In particular, myo-Ins homeostasis in cells and tissues is guaranteed at three different levels: (i) intestinal absorption and renal excretion; (ii) carrier-mediated transport from plasma/interstitial fluids into cells; and (iii) endogenous synthesis and catabolism [30]. To note, the uptake and intracellular distribution of inositol depend on different transporters. Such transporters may absorb myo-Ins and phosphate derived from the digestion of phytic acid (inositol hexakisphosphate—InsP6), which is how humans mostly consume myo-Ins. 

Bacterial phytases and phosphatases, homologous to mammalian InsP6 phosphatase, are mainly responsible for digesting dietary InsP6, releasing myo-Ins and phosphate that can be further actively absorbed by the mentioned transporters [30]. 

The impaired digestion and absorption of myo-Ins containing compounds may also correlate to altered gut microbiota composition. According to the DOGMA hypothesis, the dysbiosis of the gut microbiota can contribute to PCOS pathogenesis: an unbalanced gut microbiota may increase ovarian androgen synthesis and interfere with normal follicle development by triggering a chronic inflammatory response and a condition of insulin resistance [31,32]. Gut microbiota and its metabolites have a role in regulating inflammatory pathways, brain-gut peptide secretion, and islet β-cell proliferation [31,33,34]. Patients with PCOS exhibit significant differences in gut microbial species compared to healthy women, with increased levels of Bacteroides, Escherichia/Shigella, and Streptococcus sp. and decreased levels of *Akkermansia* and *Ruminococcaceae* sp. [31]. These microbial changes have been known to correlate with alterations in the levels of ghrelin, testosterone, and BMI. Furthermore, women with PCOS compared with healthy women have a lower α-diversity of the gut microbiome, which negatively correlates with hyperandrogenism, total testosterone, and hirsutism [35]. Gut dysbiosis also correlates to the high prevalence of obesity in patients with PCOS (80%), and changes in gut microbiota composition and enzyme secretion are responsible for impaired digestion and absorption of myo-Ins-containing compounds, thus decreasing their bioavailability in such patients [36].

Lifestyle changes and especially nutritional management are crucial in PCOS women, and a healthy diet is mandatory for counteracting metabolic alterations, with diet and exercise being the primary recourse for physicians looking to treat PCOS. Occasionally, the administration of prebiotics may help to recover the dysbiosis and low intestinal adsorption associated with this syndrome. Indeed, considering the role of the intestinal microbiota in PCOS pathogenesis, microbiota-targeted strategies are becoming increasingly relevant.

In this contest, for its prebiotic activity [37] and effectiveness in promoting the intestinal growth of specific bacterial groups [38], α-LA may induce beneficial changes in gut microbiota [39]. This has particular importance as the gut microbiota can be heavily dysregulated in patients with PCOS in terms of species and related numbers of strains. Imbalanced microbiota play a crucial role in the chronic intestinal inflammation that is a typical sign of PCOS, and they can also hamper the absorption of a wide array of substances [32,36]. 

The combination of myo-Ins with α-LA improves myo-Ins bioavailability by affecting the permeability of the intestinal tight junctions and increasing myo-Ins plasma concentration in simultaneous administration [26]. 

Small intestinal tight junctions are highly dynamic structures, rapidly responding to extracellular stimuli to maintain the integrity of the epithelial cell monolayer. Absorption enhancers may transiently modulate their permeability in order to promote the oral absorption of poorly permeable drugs.

As such, when administered in association in a single dose, α-LA led to significantly higher plasma concentrations of myo-Ins [26]. As a matter of fact, the same trend is also observed with respect to another inositol stereoisomer, such as D-chiro-inositol [40]. 

Another possible mechanism, observed in rats [27], may involve the stimulation of GLP-2 secretion from gut endocrine cells induced by α-LA hydrolysate, which can enhance small intestine absorption [28]. In specific conditions, α-LA can also work as a suitable carrier to increase the passage of molecules across the intestinal barrier [27].

In addition, other studies reported an independent role of α-LA in improving chronic intestinal inflammation in PCOS by inhibiting type 2 cyclooxygenase (COX2) and decreasing IL-6 levels [41,42], as well as boasting beneficial effects on glucose homeostasis [43].

As mentioned in the study by Montanino Oliva et al. [29], among the 14 myo-Ins-resistant patients with PCOS treated with a combination of 2 g myo-Ins plus 50 mg α-LA, twice a day, for three months, 12 of them (86%) ovulated. At the end of the treatment, their myo-Ins plasmatic levels significantly increased compared to the baseline value (35.0 ± 3.8 μmol/L vs. 17.0 ± 3.5 μmol/L) presenting similar myo-Ins levels to those of those patients who positively responded to the treatment with myo-Ins alone (38 ± 2.9 μmol/L). 

Recently, these results have been confirmed by a multicentric study [44] in which 34 insulin-resistant patients with PCOS received myo-Ins and α-LA for six months, reporting significant improvements in insulinemia, HOMA-index, and androstenedione. 

Lastly, D’Anna et al. [45] treated women with gestational diabetes mellitus with the combination of myo-Ins and α-LA, demonstrating a significant reduction of maternal insulin resistance and excessive fetal growth. Furthermore, a reduced rate of insulin treatment and preterm birth were demonstrated in patients treated for this condition.

In accordance with the published papers, our study also demonstrated that the combination of myo-Ins plus α-LA induced a greater ovulation rate and menstrual cycle improvement with respect to those women supplemented with myo-Ins as a monotherapy. In addition, this combination significantly ameliorated body weight, hyperandrogenemia, and hirsutism, suggesting that the supplementation of myo-Ins with α-LA may represent a valid therapeutic strategy not only to enhance the bioavailability of myo-Ins and their beneficial effects but also to positively restore the gut microbiota. 

Importantly, the safety of both myo-Ins and α-LA derived from their approval as “Generally Recognized as Safe” (GRAS) compounds. This reflects the widespread use of α-LA in various foodstuffs, including baby formula, where it does not cause gastrointestinal side effects. The same situation occurred in our study, where no patients reported adverse effects.

## 5. Conclusions

To our knowledge, this is the first study in the clinical context of PCOS that compares the effects of myo-Ins monotherapy vs the combination of myo-Ins plus α-LA. Overall, the association between myo-Ins and α-LA demonstrated significant improvements in reproductive and metabolic alterations in patients with PCOS. Clearly, the main weakness of the trial is the small number of patients and the short period of treatment. Studies with larger sample sizes and a long-term follow-up are needed to better assess the role of α-LA in improving the absorption and effectiveness of myo-Ins as well as to understand its role on gut microbiota, as it has become apparent that it is increasingly involved in PCOS etiopathogenesis.

## Figures and Tables

**Table 1:** Baseline characteristics of the enrolled patients.

Patients’ Characteristics	Group 1 Myo-Ins	Group 2 Myo-Ins + α-LA
Age (years)	24.9 ± 5.1	25.7 ± 5.7
BMI (kg/m^2^)	24.0 ± 5.2	26.5 ± 6.5
Menstrual cycle duration (days)	50.0 ± 22.9	51.1 ± 23.6
Prevalence of polycystic ovarian morphology %	89.5	84
FG score	6.9 ± 3.4	6.6 ± 3.2
Prevalence of hirsutism %	60	51.9
Testosterone (nmol/L)	1.6 ± 0.7	1.5 ± 0.6
Androstenedione (ng/mL)	3.9 ± 0.97	3.9 ± 1.4
DHEAS (mcmol/L)	11.01 ± 4.2	9.34 ± 3.3
Prevalence of hyperandrogenaemia %	70	55.6
HOMA index	2.4 ± 0.87	2.8 ± 2.5
Prevalence of insulin resistance %	55.6	44.4

**Table 2:** Outcomes after three months of treatment with myo-Ins alone and in combination with α-LA.

Parameters	Group 1 Myo-Ins		Group 2 Myo-Ins + α-LA		Statistics	*p* Value
Rate of ovulation (%)	47.6		79.3 *			
	Significant improvement (N patients)	No significant improvement (N patients)	Significant improvement (N patients)	No significant improvement (N patients)	Chi-square	0.019
	10	11	23	6		
Improvement of menstrual cycle duration (%)	55.6		84.0 *			
	Significant improvement (N patients)	No significant improvement (N patients)	Significant improvement (N patients)	No significant improvement (N patients)	Chi-square	0.044
	12	9	24	5		
Post-treatment menstrual cycle duration (days)	35.00 [30.00–50.00]		29.00 [28.00–30.00] **		Mann-Whitney U Test	0.00652
Weight change from baseline (kg)	0.00 [0.00–0.00]		−1.00 [−4.00–0.00] *		Mann-Whitney U Test	0.01046
Hirsutism improvement (%)	33.3		75 *			
	Significant improvement (N patients)	No significant improvement (N patients)	Significant improvement (N patients)	No significant improvement (N patients)	Chi-square	0.034
	7	14	22	7		
FG score change from baseline (pts)	0.00 [0.00–0.00]		−1.00 [0.00–2.00] *		Mann-Whitney U Test	0.0198
Hyperandrogenemia improvement (%)	46.7		55		Chi-square	0.44
Insulin resistance improvement (%)	44.4		61.5			
	Significant improvement (N patients)	No significant improvement (N patients)	Significant improvement (N patients)	No significant improvement (N patients)	Chi-square	0.361
	9	12	18	11		
HOMA index change from baseline	−0.19 [−0.89–0.14]		−0.01 [−1.04–0.87]		Mann-Whitney U Test	0.65994

* *p* < 0.05; ** *p* < 0.01.

## Data Availability

Data is contained within the article.
